# Nailfold capillary phenotypes distinguish juvenile myositis subtypes and associate with disease activity

**DOI:** 10.1002/ctm2.70789

**Published:** 2026-09-01

**Authors:** Nicholas McClellan, Li Chen, Sophia Matossian, Aurora Rynda, Kirsten Grant, Erin Janssen, J. Michelle Kahlenberg, Jessica L. Turnier

**Affiliations:** ^1^ Department of Pediatrics Division of Rheumatology University of Michigan Ann Arbor Michigan USA; ^2^ Department of Internal Medicine Division of Rheumatology University of Michigan Ann Arbor Michigan USA

**Keywords:** juvenile myositis, nailfold capillaroscopy, vasculopathy

## Abstract

**Background:**

Nailfold capillaroscopy is a non‐invasive method to visualize altered microcirculation in pediatric rheumatic disease, with potential to aid in diagnostic differentiation and disease monitoring. We used nailfold video capillaroscopy (NVC) and machine learning to identify patterns of capillaroscopic features in juvenile dermatomyositis (JDM) and associations of capillaroscopic features with clinical data.

**Methods:**

NVC features were quantified using automated neural network‐based software in 76 individuals, including 18 controls, 33 JDM, 17 childhood‐onset systemic lupus erythematosus (cSLE), and 8 overlap myositis (OM) patients. Unsupervised cluster analysis by capillaroscopic features was performed, and clinical features were characterized by cluster. Differences in capillaroscopic features between disease groups were assessed using the Kruskal–Wallis test. For JDM and OM patients, capillaroscopic and clinical feature associations were assessed using Spearman correlation. In 10 treatment‐naïve myositis patients (JDM + OM), changes in capillaroscopic features between diagnosis and 3‐month follow‐up were evaluated.

**Results:**

Cluster analysis identified three patient clusters, characterized by the presence of either branched or enlarged capillaries, or absence of these abnormalities. The “branched” cluster was most frequently assigned among JDM and OM and consisted of no controls. The majority of TIF1y+ JDM (4/5) were in the “branched” cluster. In JDM and OM, microhaemorrhage density correlated with disease duration (*r* = −.45, *p* = .0033), physician global assessment score (*r* = .6, *p* = .0004), lactate dehydrogenase (*r* = .43, *p* = .01), von Willebrand factor antigen (*r* = .51, *p* = .043), and neopterin (*r* = .69, *p* = .01). In 10 myositis patients, microhaemorrhage density decreased while capillary density increased from diagnosis to three months post‐treatment.

**Conclusions:**

A branched capillaroscopic pattern was observed more frequently in JDM and OM, particularly TIF1y+ JDM patients within our cohort. Microhaemorrhage density was the most changeable capillaroscopic feature and associated with markers of increased disease activity.

## Graphical abstract

Nailfold capillaroscopy is a non‐invasive imaging tool that can aid in the diagnosis and monitoring of juvenile myositis. Branched nailfold capillary morphology is more common in juvenile myositis patients, particularly those positive for TIF1γ+ autoantibodies. Juvenile myositis patients with more active disease demonstrate increased microhaemorrhages, which improve with treatment response.

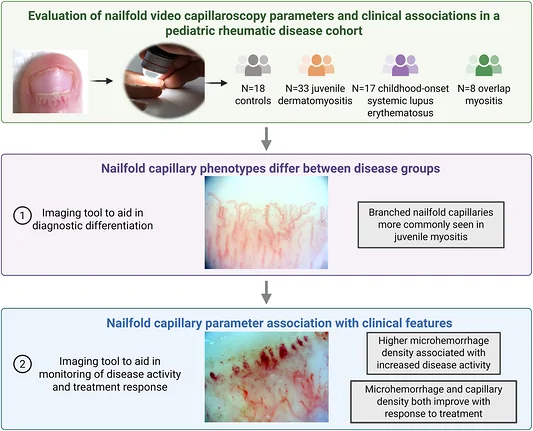

## 1 BACKGROUND

Visible alterations in nailfold capillaries (NFC) are a feature of many paediatric rheumatic diseases. Juvenile dermatomyositis (JDM) is characterized by multiorgan vasculopathy, with hallmark vascular rashes and NFC changes. NFC density has been the primary parameter assessed in JDM examination and research studies, in part due to limitations in evaluating other parameters like NFC morphology using traditional instruments, including the dermatoscope and ophthalmoscope. Advances in technology with nailfold video capillaroscopy (NVC) now allow increasingly detailed visualization and quantification of microvascular features. The ability to store images for later analysis is also a distinct advantage of NVC over analogue counterparts, allowing finer discrimination of NFC features and enabling quantitative comparisons.

A 2020 survey of paediatric rheumatologists reported widespread use of nailfold capillaroscopy, with 82% “almost always” incorporating capillaroscopy in diagnosis and 70% in monitoring, with 95% reporting only qualitative impression and no respondents using NVC.^1^ Despite the recognized importance of integrating capillaroscopy into exams, the potential utility of NVC image capture and analysis to aid diagnostic work‐up and monitoring of paediatric rheumatic diseases remains understudied. While the EULAR Study Group on Microcirculation in Rheumatic Diseases developed a standardized capillaroscopy evaluation system,^2^ this system was developed predominantly from image analysis of adult patients and those with systemic scleroderma, also with qualitative analysis of NFC features focusing on the presence of a scleroderma or scleroderma‐like pattern.^3^ Patients with myositis, compared with scleroderma, may exhibit a more changeable NFC pattern and improvement of NFC abnormalities after treatment,^4^ potentially reflecting clinical improvement and highlighting the need to further study NVC as an imaging biomarker in myositis.

Recent work utilizing NVC^5^ in children demonstrates differences in frequency of NFC abnormalities between paediatric rheumatologic conditions. JDM in particular highlights this difference, with a higher frequency of abnormal capillary shapes than other conditions evaluated, including JIA, childhood‐onset systemic lupus erythematosus (cSLE) and juvenile systemic and localized scleroderma.^5^ In fact, a recently developed artificial intelligence‐based NFC image analysis tool differentiates between JDM and controls using only NFC images.^6^ Within JDM, decreased NFC density associates with higher muscle disease activity,^7^ increased skin disease activity,^8^, ^9^ subclinical lung disease,^10^ TIF1γ autoantibodies^8^, ^9^ and chronic disease course,^11^ highlighting a potential role for NVC as an imaging biomarker of disease activity, phenotype and prognosis.

Despite accumulating evidence for the importance of NVC as an imaging tool to improve JDM care, additional studies are needed to identify consistent associations between NFC features and clinical phenotypes and guide optimal NVC use and implementation in the clinic. In this study, we utilize NVC technology and machine learning tools to (1) identify patterns of capillaroscopic findings that may distinguish between paediatric rheumatologic diseases and JDM subgroups, and (2) explore associations of NVC parameters with myositis clinical and lab features.

## 2 PATIENTS AND METHODS

### 2.1 Patient recruitment and clinical cohort characteristics

After obtaining IRB approval (HUM00230631), patients were identified with NVC performed during a Pediatric Rheumatology clinic visit at the University of Michigan C.S. Mott Children's Hospital between August 2022 and November 2024. Diagnoses assigned by treating paediatric rheumatologists were reviewed, and patients with JDM (*n* = 33), cSLE (*n* = 17), and overlap myositis (OM; *n* = 8) were included (Table 1). All JDM patients met 2017 EULAR/ACR classification criteria.^12^ All cSLE patients met 2019 EULAR/ACR classification criteria for SLE.^13^ OM patients met both 2017 EULAR/ACR classification criteria for either JDM or other JM and additionally either 2019 EULAR/ACR classification criteria for SLE (n = 6) or 2013 EULAR/ACR classification criteria for systemic sclerosis (*n* = 2) (Table S1). In cSLE patients, the absence of myositis was a clinical determination, and muscle enzymes were only checked at the time of diagnosis in *n* = 4 cSLE patients, all of whom had normal creatine kinase levels. Controls (CTL; *n* = 18) included patients without a primary rheumatologic, autoimmune or inflammatory diagnosis who had NVC performed as part of evaluation in the Pediatric Rheumatology clinic.

**TABLE 1:** Clinical characteristics of patients with juvenile dermatomyositis (JDM), childhood‐onset systemic lupus erythematosus (cSLE), overlap myositis and controls (CTL). Data are presented as mean with standard error of the mean (±SEM), unless otherwise indicated.

Column1	CTL (N = 18)	JDM (N = 33)	cSLE (N = 17)	Overlap (N = 8)
**Age at diagnosis, mean (SD); range**		9.0 (4.7); 2–19	14 (2.3); 9–17	14 (3.7); 8–17
**Age at visit, mean (SD); range**	16 (4.0); 8 ‐ 20	12 (5.1); 4–20	16 (2.8); 9–20	16 (3.1); 10–19
**Disease duration (months), mean (SD); range**		31 (31); 0–119	27 (25); 0–77	22 (38); 0–112
**Sex, *n* (%)**				
Male	5 (27.8)	7 (21.2)	2 (11.8)	0 (0.0)
Female	13 (72.2)	26 (78.8)	15 (88.2)	8 (100)
**Race/Ethnicity, *n* (%)**				
White, non‐Hispanic	13 (72.2)	17 (51.5)	6 (35.3)	2 (25.0)
Black, non‐Hispanic	3 (16.7)	8 (24.2)	8 (47.1)	3 (37.5)
Hispanic, any race	0 (0.0)	2 (6.1)	1 (5.9)	1 (12.5)
Asian, non‐Hispanic	0 (0.0)	2 (6.1)	2 (11.8)	1 (12.5)
Middle Eastern/North African, non‐Hispanic	0 (0.0)	1 (3.0)	0 (0.0)	0 (0.0)
More than 1 race	1 (5.6)	2 (6.1)	0 (0.0)	1 (12.5)
Race missing or unknown	1 (5.6)	1 (3.0)	0 (0.0)	0 (0.0)
**MSA Status, *n* (%)**				
Positive		19 (57.6)	0 (0.0)	1 (12.5)
Negative		14 (42.4)	4 (23.5)	7 (87.5)
Unknown or not done		0 (0.0)	13 (76.5)	0 (0.0)
**MSA type, *n* (%)**				
NXP‐2		8 (24.2)		0 (0.0)
TIF‐1g		5 (15.2)		1 (12.5)
MDA5		2 (6.1)		0 (0.0)
Jo‐1		1 (3.1)		0 (0.0)
Mi‐2		2 (6.1)		0 (0.0)
SAE		1 (3.1)		0 (0.0)
**MAA type, *n* (%)**				
PM/Scl	0 (0.0)	1 (3.0)	0 (0.0)	3 (37.5)
Sm	0 (0.0)	0 (0.0)	11 (64.7)	5 (62.5)
Ro	0 (0.0)	3 (9.1)^a^	7 (41.1)	3 (37.5)^a^
RNP	0 (0.0)	0 (0.0)	13 (76.5)^*^	7 (87.5)^b^
Ku	0 (0.0)	1 (3.0)	1 (5.9)	1 (12.5)
**Clinical disease activity scores, mean (SD); range**				
CDASI		3.1 (5.8); 0–30		
MMT8		136 (18.9); 93–150		
Provider global assessment (PGA)		3.3 (3.1); 0–9		
SLEDAI‐2k			7.8 (7.6); 0–22	
**Muscle enzymes, mean (SD); range**				
CK (U/L)		934.5 (2294); 53–8313		372.4 (692.8); 29–1611
LDH (U/L)		381 (299); 137–1404		333.8 (129.7); 203–454
Aldolase (IU/L)		20.3 (42.1); 3–207		10.6 (8.4); 3–22
AST (U/L)		69.2 (107); 16–556	35 (18); 17–68	104.6 (187.7); 14–529
ALT (U/L)		45.8 (59.9); 7–222	33.4 (26.1); 11–89	51.6 (46.7); 8–136
**Other labs, mean (SD); range**				
Anti‐dsDNA positive, *n* (%)			11 (64.7)	5 (62.5)
Anti‐dsDNA titre			692.8 (1778); 9.8–6669	1267.4 (1641.1); 17.3–3630.2
C3			89.6 (37.4); 34–175	90.2 (16.9); 67–109
C4			13.6 (9.6); 2–35	13.8 (9.9); 4–30
**Treatment, *n* (%)** ^c^				
On immunosuppressive therapy	0 (0.0)	24 (72.7)	15 (88.2)	7 (87.5)
Off therapy		2 (6.1)	0 (0.0)	0 (0.0)
Treatment‐naïve		7 (21.2)	2 (11.8)	1 (12.5)
On steroids		13 (39.4)	7 (41.2)	5 (62.5)
Steroid dose (mg/kg/day), mean (SD); range		3.3(8.8); 0.042–32	0.66 (0.45); 0.11–1.57	0.91 (1.2); 0.03–2.97
On MTX		11 (33.3)	2 (11.8)	1 (12.5)
On rituximab		6 (18.2)	3 (17.6)	0 (0.0)
On IVIG		15 (45.5)	0 (0.0)	0 (0.0)
On hydroxychloroquine		6 (18.2)	15 (88.2)	6 (75.0)
On mycophenolate		7 (21.2)	6 (35.3)	3 (37.5)
On belimumab		0 (0.0)	3 (17.6)	1 (12.5)

Abbreviations: ALT, alanine aminotransferase; AST, aspartate aminotransferase; CDASI, cutaneous dermatomyositis disease area and severity index score; CK, creatine kinase; cSLE, childhood‐onset systemic lupus erythematosus; CTL, control; IVIG, intravenous immunoglobulin; JDM, juvenile dermatomyositis; Jo‐1, anti‐histidyl‐tRNA synthetase; Ku, anti‐Ku antibody; LDH, lactate dehydrogenase; MAA, myositis‐associated antibodies; MDA5, melanoma differentiation‐associated gene 5; Mi‐2, anti‐Mi‐2 antibody; MMT8, manual muscle testing and a subset of eight muscles; MSA, myositis‐specific antibodies; MTX, methotrexate; NXP‐2, nuclear matrix protein 2; PGA, provider global assessment; PM/Scl, polymyositis/scleroderma; RNP, ribonucleoprotein; Ro, anti‐Ro/SSA; SAE, small ubiquitin‐like modifier activating enzyme; SEM, standard error of the mean; SLEDAI‐2k, systemic lupus erythematosus disease activity score index 2000; Sm, Smith; TIF‐1g, transcriptional intermediary factor 1‐gamma; VAS, visual analogue scale.

^a^ Two JDM and two overlap myositis patients were anti‐Ro52 positive based on Myomarker panel testing.

^b^ Only 35/58 patients (7/8 overlap myositis patients, 4/17 cSLE patients and 24/33 JDM patients) had the Myomarker panel sent for clinical testing. Of those patients that had the Myomarker panel sent, 9 patients had more specific RNP antibody subtypes to report based on Myomarker panel results (*n* = 3 cSLE patients were U1 RNP+ and *n* = 2/3 of these same cSLE patients were also weakly positive for U2 RNP, *n* = 6 overlap myositis patients were strongly U1 RNP+, and *n* = 3 of these same OM patients also displayed a weak positive to U2 RNP, and *n* = 1 of these same OM patients displayed a weak positive to U3 RNP).

^c^ Three JDM patients were each on additional therapy, including tofacitinib, adalimumab, and abatacept.

### 2.2 Clinical data collection

Retrospective chart review sourced data from electronic medical records and the capillaroscopic image database. Clinical data included standardized disease activity assessments, including Cutaneous Dermatomyositis Disease Area and Severity Index (CDASI),^14^ Physician Global Assessment (PGA),^15^ Manual Muscle Testing (MMT8)^16^ and SLE Disease Activity Index 2000 (SLEDAI‐2K).^17^ Disease activity was categorized as treatment naïve/new diagnosis if NVC was performed at diagnostic visit, active if PGA > 1 or SLEDAI‐2k>4, low disease activity/inactive if PGA≤1 or SLEDAI‐2k≤4. Of note, *n* = 4 cSLE patients had an elevated SLEDAI‐2K score due to lab abnormalities alone (elevated dsDNA titre, low complements or cytopenias). As all autoantibody testing was done for clinical testing purposes, only an extractable nuclear antibody (ENA) panel was routinely sent internally for testing at the University of Michigan. In those patients where the treating Pediatric Rheumatologist also ordered myositis‐specific autoantibody (MSA) testing, the MyoMarker 3 Profile was sent (Mayo Clinic Laboratories). Within the cohort, 35/58 patients (7/8 Overlap Myositis patients, 4/17 cSLE patients and 24/33 JDM patients) had the MyoMarker panel sent.

### 2.3 Capillaroscopic image collection

NVC was performed using the Inspectis Capillaroscope (IN‐001, Inspectis AB, Sweden) with 200x optical magnification and resolution of.84 µm per pixel, resulting in a 1.68 × 1.26 mm field of view at a standard image size of 2000 × 1500 pixels in most images. During each examination, best effort was made to obtain two images of non‐overlapping regions from digits 2–4 bilaterally. Real‐time determination of adequate quality was based on examiner assessment of ability to distinguish capillary number, shape and calibre.

### 2.4 Image review

All images were reviewed in a batch by three reviewers. A single best image was selected per finger for analysis. If no suitable images were obtained for a finger, it was excluded from calculations. Exclusions occurred if a manual capillary count could not be performed or the image was too out‐of‐focus to classify capillary structure.

### 2.5 Nailfold capillary feature quantification

**FIGURE 1:**
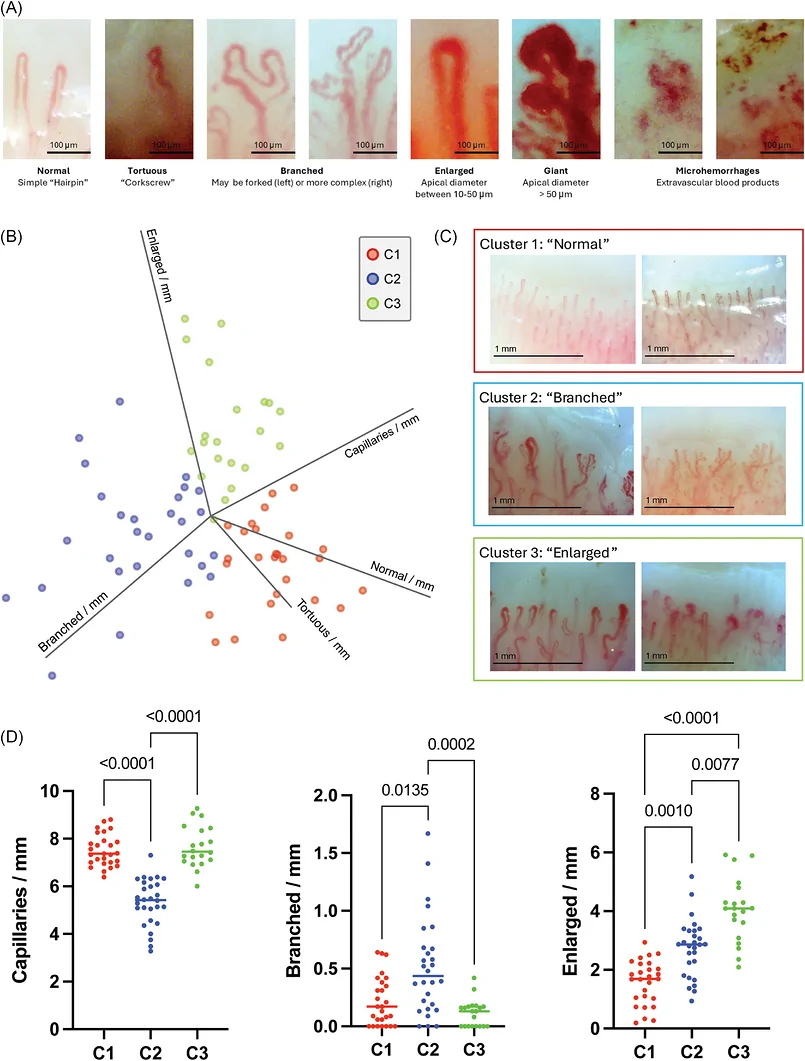
Capillaroscopic features separate patients into three identifiable clusters. (A) Example images representing capillaroscopic features evaluated. (B) Two‐dimensional plot of patients arranged by principal component analysis based on capillaroscopic features. Circles represent individual patients. Colours represent clusters assigned algorithmically based on capillaroscopic features (total capillary density (capillaries/mm), density of normal capillaries (normal/mm), density of branched capillaries (branched/mm), density of enlarged capillaries (enlarged/mm), and density of tortuous capillaries (tortuous/mm)). Axes displayed on plot provide relative contribution of primary capillaroscopic features to patient placement. (C) Representative images from each cluster. Qualitatively, cluster 1 primarily consists of simple “hairpin” loops arranged in parallel with uniform spacing. Cluster 2 demonstrates higher density of branched capillaries with increased variation in diameter. Capillaries of cluster 3 generally have a higher density of enlarged capillaries. (D) Comparison of density of capillary types by cluster, where dots represent individual patient data points and horizontal lines represent the median. Capillary types from the left: total capillaries, branched capillaries, and enlarged capillaries. Statistical significance determined by Kruskal–Wallis test.

Capillaroscopic features were detected, classified and quantified by Capillary.io, a neural network‐based system trained on images manually annotated by rheumatologists, guided by criteria developed by the EULAR study group on microcirculation with additional features reported.^2^, ^18^, ^19^, ^20^ Features classified included capillaries by dimension (normal < 20 µm, enlarged [“dilated”] 20–50 µm, giant [“megacapillary”] > 50 µm) and morphology/shape as normal or abnormal, additionally specifying branched [“ramified”] or tortuous. Regarding the utilized specific morphology terminology, branched [“ramified”] and tortuous were direct outputs from the Capillary.io software. The Capillary.io neural network‐based system classifies capillary morphology based on the fit of the capillary with a training dataset that was manually annotated by rheumatologists familiar with capillaroscopy.^18^, ^21^ Non‐capillary features identified included density (end row loops/mm; normal ≥7/mm) and microhaemorrhages. Figure 1A demonstrates examples of representative capillary features from our dataset and also highlights the morphology/shape consistent with branched and tortuous capillaries. The system generated a machine‐readable JSON file for each image. A custom Python script processed these JSON files into a human‐readable spreadsheet with summary data per patient and finger and calculated values normalized per millimetre.

### 2.6 Statistical analysis

Cluster analysis was performed using Orange 3.38.1, with unsupervised Louvain clustering and principal component analysis for two‐dimensional visual representation utilizing capillaroscopic features selected for optimal separation. Analyses for statistical significance were performed using GraphPad Prism 10.6.1. For comparisons between multiple groups, the Kruskal–Wallis test was used. Fisher's exact test identified associations between assigned clusters and disease group, duration, activity state and MSA status. Correlations were assessed using Spearman's correlation. For feature change between visits, a paired *t*‐test was used. Results with a *p*‐value < .05 after multiple comparison adjustment using Dunnett's test were considered significant. No imputation of missing data was performed. Full capillaroscopic data were available for all patients. In correlation analyses, individual pairwise analysis included only subjects with data available.

## 3 RESULTS

### 3.1 Patient cohort demographic and clinical characteristics

Table 1 summarizes clinical data for the 76 individuals at the time of NVC, including 18 CTL, 33 JDM, 17 cSLE, and 8 OM. MSAs were detected in 19/33 (57.6%) JDM, with the most common being NXP‐2 (8/33; 24.2%) and TIF1γ (5/33; 15.2%). The most common myositis‐associated autoantibody (MAA) identified in both cSLE and OM was RNP. JDM and cSLE had mild to moderate disease activity, with a mean PGA score of 3.3 in JDM and SLEDAI‐2K score of 7.8 in cSLE. More JDM relative to cSLE were treatment naïve (TN JDM: 7/33, 21.2%; TN cSLE: 2/17, 11.8%).

### 3.2 Unsupervised clustering of nailfold capillaroscopic features identifies three distinct patient clusters

Cluster analysis utilizing capillaroscopic features assigned patients to clusters based on feature similarity, identifying three distinct patient clusters (Figure 1B). Representative images of capillaroscopic features from each cluster are shown in Figure 1C with quantification in Figure 1D. Cluster 1 was defined as “normal” given normal capillary density with uniform shape and arrangement. Cluster 2 was termed “branched” given increased frequency of capillaries with abnormal morphology, including branched capillaries, and Cluster 3 was called “enlarged” given higher frequency of enlarged and/or giant capillaries. Median capillary density was lower in cluster 2 (5.42/mm) compared with both cluster 1 (7.37/mm; *p* < .0001) and 3 (7.45/mm; *p* < .0001). Branched capillaries were more frequent in cluster 2 (.44/mm) than cluster 1 (.17/mm, *p* < .0135) and cluster 3 (.13/mm, *p* = .0002). The frequency of enlarged capillaries differed between all three clusters, with more enlarged capillaries in cluster 2 (2.87/mm) versus cluster 1 (1.69/mm, *p* = .0010), though fewer than cluster 3 (4.09/mm, *p* = .0077).

### 3.3 Nailfold capillaroscopic features differ between disease groups

**FIGURE 2:**
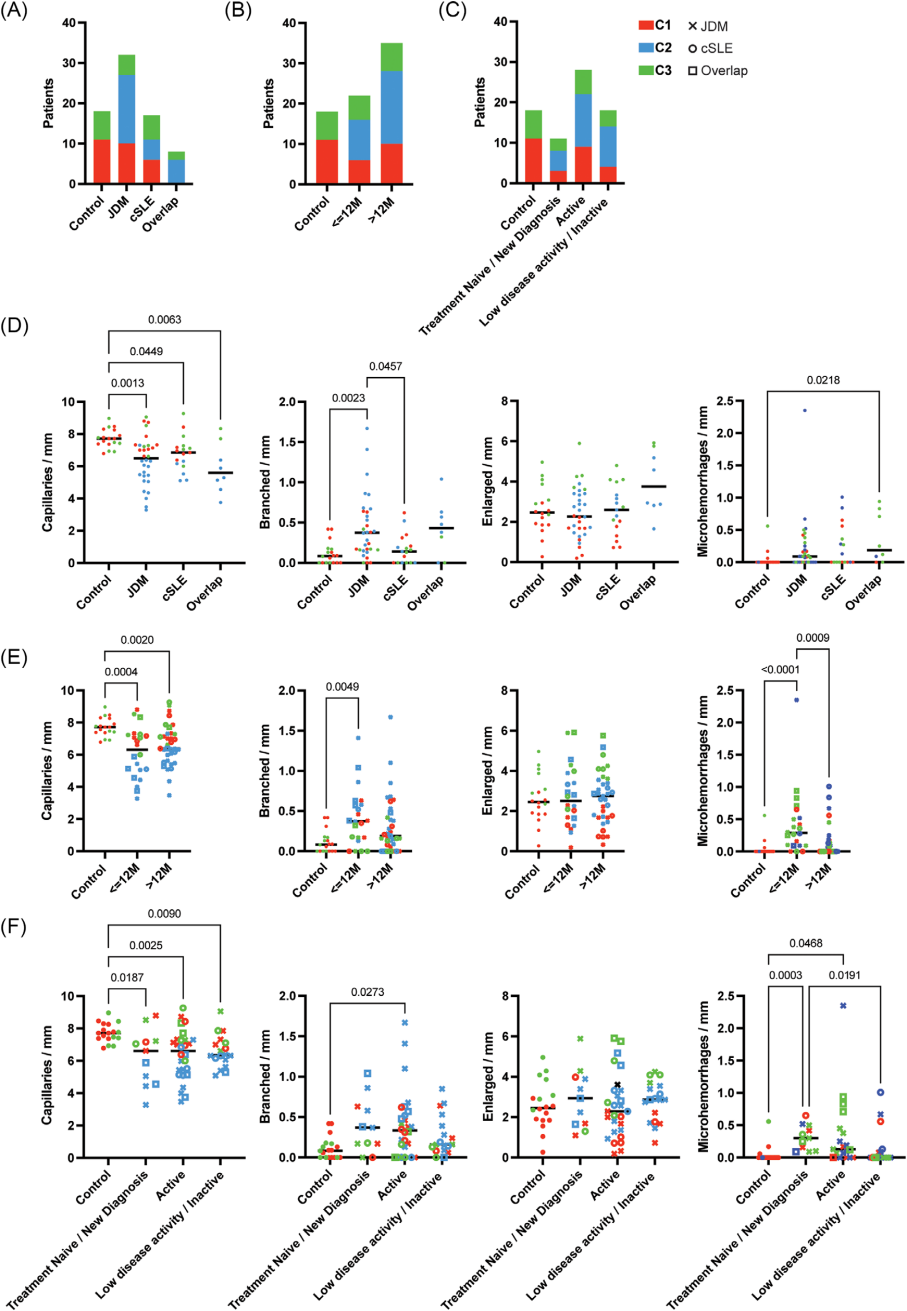
Nailfold capillaroscopic features vary by disease group, disease duration, and disease activity state. Disease groups include controls (CTL; *n* = 18), juvenile dermatomyositis (JDM; *n* = 33), childhood‐onset systemic lupus erythematosus (cSLE; *n* = 17) and Overlap Myositis (*n* = 8). (**A)** Breakdown of assigned cluster by disease group. Controls were assigned to cluster 1 or cluster 3 but not cluster 2. (**B)** Breakdown of assigned cluster by disease duration (≤12 months or >12 months). (**C)** Breakdown of assigned cluster by disease activity state (newly diagnosed/treatment naïve, active disease, low disease activity/inactive). (**D)** Comparison of nailfold capillaroscopic features by disease group. (**E)** Comparison of nailfold capillaroscopic features by disease duration. (**F)** Comparison of nailfold capillaroscopic features by disease activity state. Capillaroscopic features from left to right in (**D–F)**: total capillary density, branched capillary density, enlarged capillary density, microhaemorrhage density. Horizontal lines on plots represent the median value. Statistical significance determined by Kruskal–Wallis test.

Cluster assignment differed by diagnosis using Fisher's exact test (*p* = .0002), with most controls in “normal” cluster 1 (11/18, 61.1%), while JDM and OM were most frequently in “branched” cluster 2 (17/33 (51.5%); 6/8 (75%)) (Figure 2A). On quantitative analysis of capillaroscopic features between disease groups (Figure 2D), median capillary density differed between CTL and JDM (7.72 relative to 6.50/mm; *p* = .0013), CTL and cSLE (6.85/mm) (*p* = .0449) and CTL and OM (5.59/mm) (*p* = .0063), highlighting decreased density as a common feature across diseases. Of note, patients in cluster 2 across disease groups appeared to have the lowest capillary density (Figure 2D). As related to branched capillaries, median frequency differed between JDM (.38/mm) and CTL (.09/mm) (*p* = .0023) and also between JDM and cSLE (.14/mm) (*p* = .00457), distinguishing branched capillaries as a unique feature of JDM compared with cSLE. Of note, capillary density was negatively associated with the frequency of branched capillaries specifically in JDM and OM (*r* = −.47; *p* = .0018) (Figure S1), indicating that myositis patients with the lowest capillary density also have the most abnormal nailfold morphology.

### 3.4 Nailfold capillaroscopic features differ by disease duration and activity status

When grouping all diagnoses, cluster assignment differed by disease duration (≤12 months vs. >12 months; *p* = .0011) (Figure 2B) and disease activity status (*p* = .0044) (Figure 2C). Comparison of capillaroscopic features by time since diagnosis (Figure 2E) demonstrated no difference between median capillary density at ≤12 months (6.31/mm) or >12 months (6.39/mm), though both differed from CTL (7.72/mm; < 12 months *p* = .0004, > 12 months *p* = .0020), indicating persistently decreased NFC density even with longer disease duration. Frequency of branched capillaries was higher in patients ≤12 months from diagnosis (.38/mm) compared with CTL (.09/mm, *p* = .0049) but not at >12 months (.19/mm) from diagnosis, suggesting more capillaries with abnormal NFC morphology closer to the time of diagnosis. Microhemorrhages were more frequent before 12 months of diagnosis (.36/mm) than later (0.00/mm, *p* < .0009) and compared with CTL (0.00/mm, *p* < .0001), highlighting that patients earlier in the disease course have more microhaemorrhages.

Capillaroscopic feature evaluation by disease activity state (Figure 2F) demonstrated higher median capillary density in CTL (7.72/mm) than treatment naïve/new diagnosis (6.62/mm, *p* < .0187), active (6.62/mm, *p* < .0025), or low disease activity/inactive (6.36/mm, *p* < .0090), again showing decreased NFC density as a more longstanding feature. Branched capillaries did not differ between treatment naïve/new diagnosis (.37/mm), active (.34/mm), or low disease activity/inactive (.16/mm), but were higher in active than CTL (.09/mm, *p* < .0273). Microhemorrhages were more frequent in treatment naïve/new diagnosis (.30/mm) compared with CTL (0.00/mm, *p* = .0003) or inactive (0.00/mm, *p* = .0191), and in active (.09/mm) relative to CTL (0.00/mm, *p* < .0468), highlighting microhaemorrhage as an active disease feature.

### 3.5 Association between juvenile myositis clinical data and capillaroscopic features

**FIGURE 3:**
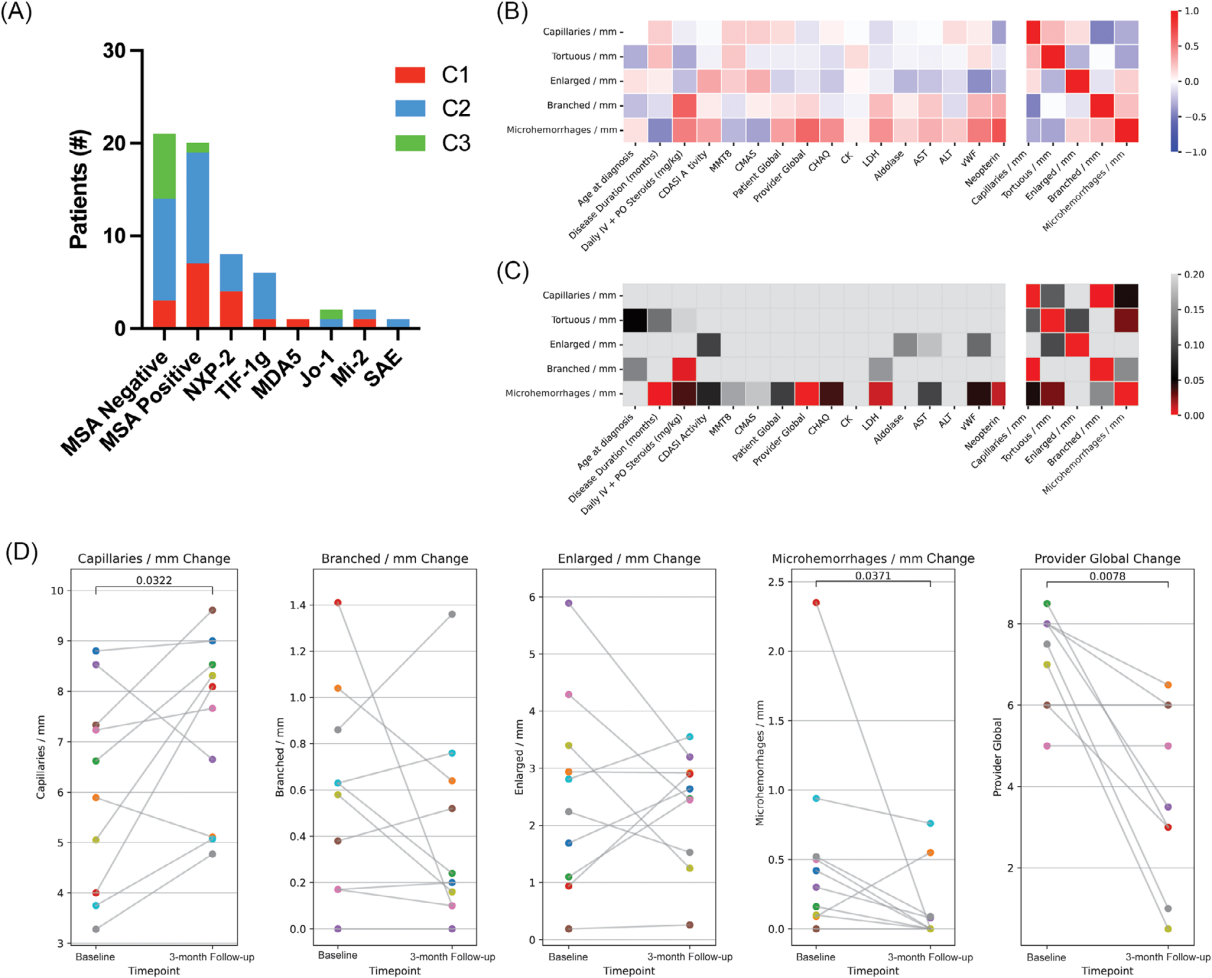
Capillaroscopic features vary with clinical and laboratory assessments in juvenile myositis (JDM and overlap myositis) patients. (**A)** Distribution of patients between clusters by MSA status. (**B)** Correlation matrix demonstrating correlation of clinical, lab, and capillaroscopic features in juvenile myositis patients. Spearman coefficient reported for each pairwise comparison by colour from −1.0 (blue) to 1.0 (red). (**C)** Correlation matrix demonstrating correlation of clinical, lab, and capillaroscopic features in juvenile myositis patients. Spearman significance reported for each pairwise comparison by colour, with increasing black shade for.2 > *p* > .05 and increasing red for *p* < .05. (**D)** Longitudinal changes between diagnostic/baseline and 3‐month follow‐up visit of the same patients (*n* = 10; including *n* = 8 JDM and *n* = 2 overlap myositis patients). Capillaroscopic features from left to right: total capillary density, branched capillary density, enlarged capillary density, microhaemorrhage density, Physician Global Assessment (PGA) score. Statistical significance determined by paired *t*‐test.

JDM and OM patients were combined for further analysis. There was no difference in cluster assignment between MSA+ and MSA‐ patients; however, most TIF1γ+ JDM patients were in cluster 2 (4/5; 80%) (Figure 3A). The one TIF1γ+ OM patient was also in cluster 2, highlighting that myositis patients with TIF1γ autoantibodies have the most abnormal NVC phenotype based on morphological changes within our single‐centre cohort. NXP2+ JDM had the highest frequency of a “normal” NVC phenotype, with 4/8 or 50% in cluster 1.

Associations between clinical data and capillaroscopic features are displayed in correlation matrices for strength (Figure 3B) and statistical significance (Figure 3C). Microhaemorrhage density demonstrated the strongest association with clinical features and associated with PGA (*r* = .6, *p* = .0004), LDH (*r* = .43, *p* = .01), vWF antigen (*r* = .51, *p* = .043) and neopterin (*r* = .69, *p* = .01), also approaching significance with CDASI activity (*r* = .36, *p* = .052).

### 3.6 Changes in capillaroscopic features in juvenile myositis patients after diagnosis and treatment initiation

In a subset of newly diagnosed, treatment‐naive JDM and OM (*n* = 10) with NVC performed at baseline and 3‐month follow‐up (Table S2), changes in capillaroscopic features between visits were evaluated (Figure 3D). PGA scores improved between visits (median PGA of 7.75 at baseline and 4.25 at follow‐up; *p* = .0078). After treatment initiation, patients had improvement in both median capillary density (6.26/mm at baseline to 7.88/mm at follow‐up; *p* = .0322) and median microhaemorrhage density (.36/mm at baseline to 0.00/mm at follow‐up; *p* = .0371).

## 4 DISCUSSION

By utilizing NVC technology and machine learning, we identified a nailfold capillaroscopy phenotype defined by abnormal, branched morphology characterizing juvenile myositis patients. While decreased NFC density defined all disease groups, branched nailfold morphology distinguished JDM as compared with cSLE and controls. Among MSA subtypes reflected in our cohort, most TIF1γ‐positive JDM demonstrated a branched morphology NVC phenotype, highlighting pronounced NFC abnormalities in this MSA subgroup. Microhaemorrhage density was associated with the most clinical variables in our cross‐sectional juvenile myositis cohort, including global disease activity and neopterin, LDH and vWF antigen. Microhaemorrhage density was also the most changeable NFC parameter over time in a subset of 10 juvenile myositis patients in our cohort, showing improvement from diagnosis to 3‐month follow‐up, indicating potential utility as an imaging biomarker.

Branched capillary morphology distinguished JDM from cSLE and controls, highlighting that unique capillary morphology is associated with myositis and may reflect underlying pathogenesis. This finding was also reported by Melsens et al., where more abnormal shapes were identified in JDM and mixed connective tissue disease (MCTD) compared with other paediatric rheumatic diseases, including scleroderma.^5^ In another paediatric study comparing nailfold capillaroscopy findings across paediatric rheumatic diseases, children with JDM and OM were grouped with juvenile systemic scleroderma (jSSc) and MCTD, and this group had the most capillary morphology abnormalities and also the highest capillary loop width, potentially reflecting extent of branching, even as compared with cSLE, JIA, primary Raynaud's and primary vasculitis.^22^ Similarly, adult dermatomyositis (DM) patients have been noted to have more frequent branched or ramified capillaries, even as compared with adult SSc patients^23^ and other forms of IIM.^24^ DM patients also appear to have a distinct combination and progression of capillaroscopy findings over time as compared with SSc, with the more common early and often unique presence of giant‐ramified capillaries and the recovery of capillary density over time.^23^ In contrast, SSc patients more commonly have a defined progression of nailfold capillary changes over time with less variability and changeability, with early presence of giant capillaries and microhaemorrhage and progressive capillary loss and later presence of ramified capillaries, as reflected in the current standardized definition of early, active and late scleroderma patterns by the EULAR Study Group on Microcirculation in Rheumatic Diseases.^2^ This suggests that the combination and progression of nailfold capillary changes may reflect differences in disease‐specific pathogenesis that could lend insight into precision medicine in rheumatic disease. As has been suggested by others,^23^ it is important that a JDM/DM pattern be considered in capillaroscopy classification, and abnormal morphology would be a key capillary parameter to include in this new definition.

A branched capillaroscopic pattern was also observed more frequently in TIF1γ+ JDM patients in our study, indicating that frequency of abnormal capillary morphology may associate with specific MSAs and that additional study of morphology may also improve understanding of disease heterogeneity. In adult DM, an active scleroderma pattern has been observed more commonly in TIF1γ+ DM.^24^ TIF1γ+ JDM patients have also been described to have a higher apical loop diameter as well as a trend towards increased neoangiogenesis.^25^ We also found JDM patients with more branched capillaries to have lower density, and Bica et al. also found that TIF1γ, compared with NXP2 and MDA5 autoantibodies, was associated with more capillary loss.^9^ Pachman et al also demonstrated that TIF1γ+ JDM exhibit more capillary loss,^8^ supporting that MSA status may impact the degree of vasculopathy and nailfold capillaroscopy findings.

Nailfold capillary morphology can be a challenging parameter to standardize in clinical assessments, with overall low inter‐rater reliability,^26^ and also with multiple different terms historically used to describe the structural capillary changes visualized in rheumatic disease. The nomenclature that has been used to describe capillaries with abnormal morphology in JDM includes branched or ramified, bushy and bizarre.^9^, ^25^ Most recently, the EULAR Study Group on Microcirculation in Rheumatic Diseases developed a simplified definition of “abnormal” capillary morphology to facilitate translatability and application across all rheumatic diseases,^2^, ^27^ although within JDM and myositis, it may still be helpful to develop more specific terminology as related to morphology to capture disease heterogeneity and define disease‐specific imaging biomarkers within myositis. The use of machine learning tools and automated analysis to classify nailfold morphology may help to further develop insight into the underlying biological meaning of specific nailfold changes.^18^, ^21^

We identified microhaemorrhage density to decrease with improvement in global disease activity, suggesting that monitoring microhaemorrhage density may aid in disease activity monitoring. In a recent assessment of NVC parameters in adult myositis paired to peripheral blood interferon‐stimulated gene signatures and serum cytokines, microhaemorrhage density associated with interferon activation, supporting that microhaemorrhage may be a helpful parameter to assess in disease activity monitoring.^28^ While NFC density improved over time in our treatment‐naïve myositis patients in our study, it did not associate with disease activity in our larger, cross‐sectional myositis cohort, suggesting that NFC density may not be as readily changeable in chronic disease. NFC density has previously been associated with chronic JDM disease course.^11^ As related to disease activity, studies have varied in organ‐specific associations, with more studies linking decreased NFC density to increased skin disease activity^8^, ^9^ compared with higher muscle disease activity.^7^ Further study is needed to determine whether NFC parameters, such as microhaemorrhage density, may predict disease flare, and the timing of NFC parameter changes relative to symptoms of flare. This will translate to the identification of specific NVC parameters in JDM that can best serve as novel imaging biomarkers of disease activity and aid in optimizing clinical outcomes for children with JDM.

While our study contributes to understanding NVC findings as related to JDM clinical characteristics, we acknowledge that several factors may limit the strength of our conclusions. The cohort was from a single centre, with heterogeneous organ manifestations and disease activity and small numbers of patients in each MSA subgroup. Specifically as related to findings by MSA subgroup, we had a limited number of JDM patients that were TIF1γ+ (*n* = 5), thereby dampening the generalizability of these conclusions and necessitating a larger cohort of TIF1γ+ to confirm these findings in the future. As related to cross‐sectional analysis by disease duration, we are also limited by differences in treatment choices and timing of treatment initiation across patients. Bias may be introduced through image selection at several stages, including acquisition and review, and may reflect challenges in obtaining images related to patient‐related factors. Though automated quantification yields reproducible identification of capillary features, the neural network‐based method introduces potential biases from underlying training data. However, prior studies suggest the model utilized has good agreement on capillary feature classification between human reviewers.^18^ Further, our conclusions related to abnormal morphology are also specific to branched or ramified capillaries as classified by the Capillary.io neural network‐based system, and larger studies are needed to assess NVC morphology more broadly in myositis.

## 5 CONCLUSIONS

Overall, our study adds to the literature by confirming branched NFC morphology as a distinguishing feature of myositis and TIF1γ+ JDM. We highlight microhaemorrhage density as a more changeable NFC feature that associates with disease activity in myositis. Further study of longitudinal NVC changes in a multicentre JDM cohort in relation to disease activity, flare and prognosis, as well as additional characterization of capillary morphology and branching, represents necessary next steps to optimize nailfold capillaroscopy use in JDM and improve patient care.

## AUTHOR CONTRIBUTIONS

Conceptualization: Jessica L. Turnier, Nicholas McClellan, and J. Michelle Kahlenberg. Data curation and visualization: Jessica L. Turnier, Nicholas McClellan, Li Chen, Sophia Matossian, and Aurora Rynda. Formal analysis: Nicholas McClellan, Li Chen. Funding acquisition: Jessica L. Turnier. Investigation: Jessica L. Turnier, Nicholas McClellan, Li Chen, Sophia Matossian, and Aurora Rynda. Methodology: Jessica L. Turnier, Nicholas McClellan, and Li Chen. Project administration: Jessica L. Turnier, Aurora Rynda, and J. Michelle Kahlenberg. Software: Nicholas McClellan and Li Chen. Supervision: Jessica L. Turnier, Erin Janssen, and J. Michelle Kahlenberg. Validation: Jessica L. Turnier and Li Chen. Writing—original draft: Jessica L. Turnier, Nicholas McClellan, Li Chen, Aurora Rynda, and Kirsten Grant. Writing—review and editing: Jessica L. Turnier, Nicholas McClellan, Li Chen, Sophia Matossian, Aurora Rynda, Kirsten Grant, Erin Janssen, and J. Michelle Kahlenberg.

## CONFLICT OF INTEREST STATEMENT

Jessica L. Turnier served on an advisory panel for Cabaletta Bio. J. Michelle Kahlenberg has received grant support from Q32 Bio, Celgene/Bristol‐Myers Squibb, Ventus Therapeutics, Rome Therapeutics, and Janssen. J. Michelle Kahlenberg has served on advisory boards for AstraZeneca, Biogen, Bristol‐Myers Squibb, Eli Lilly, EMD Serono, Exo Therapeutics, Gilead, GlaxoSmithKline, Integer Bio, Aurinia Pharmaceuticals, Rome Therapeutics, Synthekine, Seismic Therapeutics, Vivideon, and Ventus Therapeutics. Erin Janssen is on advisory panels for SOBI and Incyte. The remaining authors declare no conflict of interest.

## ETHICS STATEMENT

All activities in this study were approved by the University of Michigan Internal Review Board (HUM00230631).

## Supporting information




Supporting File 1

Supporting File 2

**Table S1**. Clinical characteristics and classification of overlap myositis patients (*n* = 8). Of note, 7/8 patients had the Myomarker panel sent. For the Myomarker panel, autoantibody results were interpreted as follows: <20 U; weak positive, 20–39 U; moderate positive, 40–80; strong positive, >80 U. Anti‐double‐stranded DNA (dsDNA) antibodies were considered within the normal range at <27 IU/mL. Autoantibodies identified by the extractable nuclear antibody (ENA) panel (indicated by an asterisk [*]) were reported qualitatively and were not further titrated; therefore, quantitative antibody levels were not available. Abbreviations: ANA, antinuclear antibody; JDM, juvenile dermatomyositis; JM; juvenile myositis; SLE, systemic lupus erythematosus; SLEDAI‐2k, Systemic Lupus Erythematosus Disease Activity Score Index 2000; dsDNA, anti‐double stranded deoxyribonucleic acid; MSA, myositis‐specific antibodies; MAA, myositis‐associated antibodies; TIF‐1g, transcriptional intermediary factor 1‐gamma; PM/Scl, polymyositis/scleroderma; Sm, Smith; Ro, Sjögren syndrome‐related antigen A (Ro) antibody; SS‐A 52kd, Sjögren syndrome‐related antigen A 52kd antibody; RNP, ribonucleoprotein; Ku, anti‐Ku antibody; Pl‐7, threonyl‐transfer RNA synthetase; Scl‐70, scleroderma‐70; La, Sjögren syndrome‐related antigen B (LA) antibody
**Table S2**. Clinical and laboratory characteristics of JDM and overlap myositis patients (*n* = 10) at baseline (BL) and follow‐up (FU) visits. Laboratory values were obtained within a month of the corresponding office visit. Creatine kinase (CK), lactate dehydrogenase (LDH) and aldolase were all missing data points from three patients (*n* = 3). No patients were on rituximab, mycophenolate or belimumab at the follow‐up visit. *The variable “Daily IV + PO Steroids (mg/kg)” was calculated as the average daily total (IV+PO) prednisone equivalent dose over the past 28 days.
**Figure S1**. Correlation between capillaroscopic features by diagnosis (Juvenile Myositis (JDM + Overlap Myositis), cSLE, CTL). (**A)** Correlation matrix demonstrating correlation of capillaroscopic features in juvenile myositis patients. Spearman coefficient reported for each pairwise comparison by colour from −1.0 (blue) to 1.0 (red). (**B)** Correlation matrix demonstrating correlation of capillaroscopic features in juvenile myositis patients. Spearman significance reported for each pairwise comparison by colour, with increasing black shade for.2 > *p* > .05 and increasing red for *p* < .05. **C)** Correlation matrix demonstrating correlation of capillaroscopic features in cSLE patients. Spearman coefficient reported for each pairwise comparison by colour from ‐1.0 (blue) to 1.0 (red). (**D)** Correlation matrix demonstrating correlation of capillaroscopic features in cSLE patients. Spearman significance reported for each pairwise comparison by colour, with increasing black shade for.2 > *p* > .05 and increasing red for *p* < .05. (**E)** Correlation matrix demonstrating correlation of capillaroscopic features in control patients. Spearman coefficient reported for each pairwise comparison by colour from −1.0 (blue) to 1.0 (red). (**F)** Correlation matrix demonstrating correlation of capillaroscopic features in control patients. Spearman significance reported for each pairwise comparison by colour, with increasing black shade for.2 > *p* > .05 and increasing red for *p* < .05.

## Data Availability

Data are available from authors upon reasonable request.
