# Supplementary material for: Nailfold capillary phenotypes distinguish juvenile myositis subtypes and associate with disease activity
Source: Clin Transl Med. 2026 Sep 1;16(9):e70789. doi: 10.1002/ctm2.70789 (PMC13535121; doi:10.1002/ctm2.70789)
Supplement: Supplementary file 1 [file CTM2-16-e70789-s001.pdf]

	BL (n=10)	FU (n=10)
Demographics		
**Age at diagnosis, mean (SD); range**	10 (5.12), 4 - 19	
**Time since diagnosis (months), mean (SD); range**	0.30 (0.67); 0 - 1	3.6 (2.37); 1 - 7
**Sex, n (%)**		
**Male**	2 (20.0)	
**Female**	8 (80.0)	
**Race/Ethnicity, n (%)**		
**White, non-Hispanic**	5 (50.0)	
**Black, non-Hispanic**	1 (10.0)	
**Hispanic, any race**	1 (10.0)	
**Asian, non-Hispanic**	1 (10.0)	
**Middle Eastern/North African, non-Hispanic**	0 (0.0)	
**More than 1 race**	1 (10.0)	
**Race missing or unknown**	1 (10.0)	
Disease Group		
**JDM**	8 (80.0)	
**Overlap**	2 (20.0)	
MSA Status, n (%)		
**Positive**	7 (70.0)	
**Negative**	3 (30.0)	
**Unknown or not done**	0 (0.0)	
**MSA type, n (%)**		
**NXP-2**	6 (60.0)	
**Jo-1**	1 (10.0)	
**MAA type, n (%)**		
**PM/Scl**	2 (20.0)	
**Ro**	1 (10.0)	
**RNP**	4 (40.0)	
**Unknown or not done**	1 (10.0)	
Clinical disease activity scores, mean (SD); range		
**CDASI activity**	7.3 (11.5); 0 - 30, n=6	2.7 (3.6); 0 - 8, n=7
**MMT8**	114 (22.9); 93-150, n=5	129 (16.9); 104 - 150, n=5
**Provider Global Assessment (PGA), VAS**	7.2 (1.2); 5-8.5, n=10	4.2 (2.3); 0.5-7, n=10
NFC Capillary Parameters, mean (SD); range		
**Capillaries / mm**	6.05 (1.98); 3.28- 8.80	7.28 (1.77); 4.77-9.00
**Branched / mm**	0.59 (0.44); 0.00 - 1.41	0.41 (0.42); 0.00 - 1.36
**Enlarged / mm**	2.55 (1.71); 0.19 - 5.89	2.32 (1.01); 0.26 - 3.55
**Microhemorrhages / mm**	0.54 (0.70); 0.00 - 2.35	0.15 (0.27); 0.00 - 0.76
Labs		
**Muscle enzymes, mean (SD); range**		
**CK (U/L)**	1183.9 (2565.87); 29 - 8318	104.1 (95.49); 38 - 277
**LDH (U/L)**	485.6 (216.57); 242 - 1011	333.9 (99.06); 256 - 526
**Aldolase (IU/L)**	14.8 (13.10); 4-49	7.0 (2.38); 5 - 11
**AST (U/L)**	111.1 (163.75); 22 - 556	31.3 (17.94); 14 - 75
**ALT (U/L)**	51.9 (60.90); 8 - 206	29.5 (32.10); 7-115
Treatment, n (%)		
**On immunosuppresive therapy**		9 (90.0)
**On steroids**		8 (80.0)
***Daily IV + PO Steroids (mg/kg), mean (SD); range**		2.26 (1.63); 0.53 - 5.95
**Daily PO dose (mg), mean (SD); range**		27.56 (18.02); 8.0 - 60.0
**On MTX**		6 (60.0)
**On CellCept**		2 (20.0)
**On IVIG**		5 (50.0)
**On hydroxychloroquine**		4 (40.0)
