# Supplementary material for: Nailfold capillary phenotypes distinguish juvenile myositis subtypes and associate with disease activity
Source: Clin Transl Med. 2026 Sep 1;16(9):e70789. doi: 10.1002/ctm2.70789 (PMC13535121; doi:10.1002/ctm2.70789)
Supplement: Supplementary file 2 [file CTM2-16-e70789-s003.pdf]

Overlap Myositis Subject ID	Meets 2017 EULAR/ACR classification criteria for JDM or other JM	Meets 2019 EULAR/ACR classification criteria for SLE	Meets 2013 EULAR/ACR classification criteria for systemic sclerosis	Presence of sclerodactyly?	Presence of Raynaud's?	Presence of pathognomonic JDM rash?	Presence of abnormal nailfold capillaries?	Other key clinical features in addition to myositis (i.e arthritis, nephritis, cytopenias, lung disease, hypocomplementemia)	MSA present?	MAA present?	dsDNA present?
1	Yes	Yes	No	No	Yes	No	Yes	Yes; arthritis, cytopenias, rash, oral ulcers	Yes (TIF1-g, 41)	Yes: ANA (>1:2560), U1 RNP (178), Sm (100.3), SmRNP*,U3 RNP (weak positive), PM/Scl (132), Ro*	Yes (2353.1 IU/mL)
2	Yes	Yes	No	No	Yes	No	Yes	Yes; arthritis, rash	No	Yes: ANA (>1:2560), U1 RNP (166), U2 RNP (weak posiitve), SmRNP*, Scl-70*	No
3	Yes	Yes	No	No	Yes	No	Yes	Yes; arthritis, cytopenias, hypocomplementemia	No	Yes: ANA(>1:2560), U1 RNP (147), U2 RNP (weak positive), Sm*	Yes (42.9 IU/mL)
4	Yes	Yes	No	No	No	Yes	Yes	Yes; arthritis, cytopenias, rash, nephritis, hepatitis	No	Yes: ANA (>1:2560), U1 RNP (105), SmRNP*, Pm/Scl (30), Sm*, SS-A-52kd (198), La*	Yes (3630.2 IU/mL)
5	Yes	Yes	No	No	No	Yes	Yes	Yes; arthritis, cytopenias, rash, oral ulcers, alopecia, hypocomplementemia	No	Yes: ANA*, U1 RNP (83), SmRNP*	Yes (293.5 IU/mL)
6	Yes	No	Yes	Yes	Yes	Yes	Yes	Yes; calcinosis, rash	No	Yes: ANA (>1:2560), Pm/Scl (173)	No
7	Yes	No	Yes	Yes	Yes	No	Yes	Yes; arthritis	No	Yes: ANA*, RNP*, SmRNP*, Sm*	No
8	Yes	Yes	No	No	Yes	Yes	Yes	Yes; arthritis, cytopenias, rash, nephritis	No	Yes: ANA (1:1280), U1 RNP (174), U2 RNP (weak positive), SmRNP*, Sm*, SS-A 52kd (176)	Yes (3366.3 IU/mL)
